# The Hsp70–Hsp90 go-between Hop/Stip1/Sti1 is a proteostatic switch and may be a drug target in cancer and neurodegeneration

**DOI:** 10.1007/s00018-021-03962-z

**Published:** 2021-10-22

**Authors:** Kaushik Bhattacharya, Didier Picard

**Affiliations:** Département de Biologie Cellulaire, Université de Genève, Sciences III, 1211 Genève 4, Switzerland

**Keywords:** Molecular chaperone, Proteostasis, Protein folding, Degradation, Proteasome, Aggregation, Stress response, Aging

## Abstract

The Hsp70 and Hsp90 molecular chaperone systems are critical regulators of protein homeostasis (proteostasis) in eukaryotes under normal and stressed conditions. The Hsp70 and Hsp90 systems physically and functionally interact to ensure cellular proteostasis. Co-chaperones interact with Hsp70 and Hsp90 to regulate and to promote their molecular chaperone functions. Mammalian Hop, also called Stip1, and its budding yeast ortholog Sti1 are eukaryote-specific co-chaperones, which have been thought to be essential for substrate (“client”) transfer from Hsp70 to Hsp90. Substrate transfer is facilitated by the ability of Hop to interact simultaneously with Hsp70 and Hsp90 as part of a ternary complex. Intriguingly, in prokaryotes, which lack a Hop ortholog, the Hsp70 and Hsp90 orthologs interact directly. Recent evidence shows that eukaryotic Hsp70 and Hsp90 can also form a prokaryote-like binary chaperone complex in the absence of Hop, and that this binary complex displays enhanced protein folding and anti-aggregation activities. The canonical Hsp70-Hop-Hsp90 ternary chaperone complex is essential for optimal maturation and stability of a small subset of clients, including the glucocorticoid receptor, the tyrosine kinase v-Src, and the 26S/30S proteasome. Whereas many cancers have increased levels of Hop, the levels of Hop decrease in the aging human brain. Since Hop is not essential in all eukaryotic cells and organisms, tuning Hop levels or activity might be beneficial for the treatment of cancer and neurodegeneration.

## Introduction

The 90 kDa and 70 kDa heat shock proteins (Hsp) Hsp90 and Hsp70, respectively, are ATP-dependent molecular chaperones essential for eukaryotic life. Hsp70 is composed of two main domains: the N-terminal nucleotide-binding domain (NBD), and the C-terminal substrate-binding domain (SBD), which associates with substrate proteins by recognizing short exposed hydrophobic stretches in the initial stages of substrate folding [1–4]. Hsp70-driven protein folding is a concerted process with Hsp40 and a nucleotide exchange factor (NEF) [1, 2, 4]. Moreover, Hsp70 prevents protein aggregation, solubilizes aggregated proteins, and assists in clearing misfolded proteins and aggregates primarily via chaperone-mediated autophagy (CMA) [1, 2, 5–7].

The Hsp90 chaperone system works downstream of the Hsp70 chaperone system and is responsible for the final stages of folding and maturation of substrate proteins (discussed later in detail). There are two cytosolic isoforms of Hsp90 identified in mammals, Hsp90α and Hsp90β, and in budding yeast, Hsp82 and Hsc82, the inducible and constitutively expressed isoforms of Hsp90, respectively [8–10]. Hsp90 paralogs are found in mitochondria (Trap1) and the endoplasmic reticulum (Grp94 or Gp96) along with organelle-specific paralogs of Hsp70 (Mortalin in mitochondria and Grp78 in the endoplasmic reticulum) [5, 10]. Hsp90 isoforms exist as dynamic homodimers. Each protomer of the homodimer is composed of three highly conserved domains: the N-terminal domain (NTD) involved in nucleotide (ATP) binding and hydrolysis, the middle domain (MD), which contributes the catalytic lysine residue for the ATPase activity and is responsible for binding many of the substrates, and the C-terminal domain (CTD) responsible for dimerization [9–12]. The substrate proteins are collectively called Hsp90 “clients” [4, 9, 11–14]. The current set of Hsp90 clients includes kinases, transcription factors, including steroid receptors, and many other proteins, sharing no common features in terms of sequence or structure [9, 10, 12]. A comprehensive and continuously updated list of Hsp90 clients and other interactors is available at https://www.picard.ch/downloads/Hsp90interactors.pdf [15].

Hsp90-mediated client protein folding or assembly is a highly regulated process, which requires the sequential association and release of co-chaperones [9, 10, 12]. One of these co-chaperones is Hsp70–Hsp90 organizing protein (Hop), encoded by the gene *STIP1* in vertebrates and *STI1* in yeast. Justifying its name, Hop is an adaptor protein between Hsp70 and Hsp90 [10, 16]. It is proposed that Hop facilitates the client transfer from the Hsp70 to the Hsp90 chaperone systems and thereby promotes the protein folding process.

This review will discuss the significant discoveries around Hop in the last three decades and the progress in the understanding of its molecular functions and physiological relevance. We mainly focus on the role and regulation of Hop and its impact on the Hsp70–Hsp90 molecular chaperone machines and protein homeostasis (proteostasis). Moreover, we propose the possible implications of modulating Hop as a therapeutic target in cancer and neurodegenerative diseases. A continuously updated literature overview on Hop is available at https://www.picard.ch/downloads/Hopfacts.pdf.

## Discovery, expression, and localization of Hop

The gene for Hop was first discovered in budding yeast as a stress-inducible gene, hence named *STI1* (or Sti1 for the protein) in that species [17]. Consistent with its heat shock-induced overexpression, the core heat-shock element (HSE) TTC-GAA-TTC-GTA was found at position − 241 of the promoter region of the *STI1* gene [17, 18]. The yeast *STI1* gene was found to encode a polypeptide of 589 amino acid residues with a calculated molecular mass of 66.246 kDa [17]. However, the experimental molecular mass of Sti1 was determined to be approximately 73 to 75 kDa by SDS-PAGE analysis [17]. Two-dimensional gel electrophoresis revealed four isoforms with isoelectric points from 5.75 to 6.05 in yeast [17]. This finding indicated that the endogenous Sti1/Hop protein might be post-translationally modified, for example by differential phosphorylation. In this context, it is noteworthy that Hop is also known as stress-induced phosphoprotein 1 (Stip1) in mammals. Hop was first identified and characterized in a human cell line as an SV40 transformation-induced protein [19]. Human Hop is a 543-amino acid protein with a calculated molecular mass of 62.6 kDa [19]. Sequence alignments revealed 42% amino acid sequence identity between the orthologs of Hop from humans and yeast [19].

Although Sti1/Hop was discovered as a stress-induced gene, the mechanism of this regulation is not well understood. The existence of an HSE in the promoter sequence of *STI1* would allow the binding of Hsf1, the master transcriptional regulator of the heat-shock response (HSR). Inhibition of Hsp90 in human gastric cancer cells can also induce the HSR by activating Hsf1, which is then recruited to the promoters of *STIP1* and other target genes, including *BAG3, HSPA1A, HSPA1B, FKBP4, and UBB* [20]. Alternatively, under hypoxic stress in primary cortical cultures from mice, transcriptional activation of *STIP1* was shown to be driven by HIF1α, the master regulator of the hypoxic response [21]. A hypoxia response element (HRE) was found at positions -60 to − 63 of the promoter and thought to be responsible for its expression during oxygen deprivation in mammalian cells [21]. Furthermore, the HRAS oncogene and mutant p53 positively regulate the transcription of *STIP1*, whereas wild-type p53 inhibits it [22]. Although the expression of Hop under stress conditions has been explained, how it is regulated under normal physiological conditions remains to be investigated. Moreover, the translational control of Hop and the functions of the 5′- and 3′-UTRs of its mRNA need to be studied under both normal physiological and stress conditions.

Hop is primarily localized in the cytosol [23]. However, heat-shock treatment and hydroxyurea-induced G1/S cell cycle arrest promote nuclear localization of Hop [24, 25]. These findings suggest that Hop shuttles between the cytosol and the nucleus. The post-translational modifications regulate the subcellular localization of Hop. There is evidence that there are two distinct phosphorylation sites for murine Hop, S189 and T198, which are phosphorylated by casein kinase II and Cdk1, respectively [26]. Intriguingly, whereas S189 phosphorylation promotes nuclear localization of murine Hop, T198 phosphorylation restrains it in the cytosol [24]. Since a cell cycle-specific kinase (Cdk1) phosphorylates Hop, it is proposed that Hop can differentially localize in cells depending on the cell cycle phases. This further raises the possibility of cell cycle phase-specific functions of Hop, an idea that is supported by the observation that a Hop knockdown or knockout in human cancer cells leads to a slowdown in G1 [27–29]. Interestingly, the phosphorylation sites of Hop are conserved between its murine and human orthologs. Although these phosphorylation sites are well-characterized for murine Hop, functions of these sites are yet to be described for the human ortholog, which only differs by 14 amino acids. Mammalian Hop can also be SUMOylated by PIAS1, an E3 SUMO ligase [30]. Interestingly, the interaction with PAIS1 promotes the nuclear localization and retention of Hop independently of SUMOylation [30].

Apart from the cytosol and the nucleus, a fraction of the intracellular Hop resides in the Golgi apparatus [19] and at the cell membrane [31, 32]. Many cell types can also secrete Hop. Glial cells and astrocytes secret Hop as a neurotrophic ligand, which triggers prion protein (PrP^c^) signaling in neurons with neuroprotective effects [32–35]. Hop was also reported to be secreted by hepatocellular, ovarian, brain, and gastric carcinoma cells and tissues [36–40]. For ovarian and gastric carcinoma, secreted Hop was proposed to be a diagnostic and prognostic marker [38, 39, 41]. Additionally, Hop is present in extracellular vesicles or exosomes released by mouse embryonic stem cells and astrocytes, and is thought to play a role in intercellular communication [42, 43]. Hop, together with Hsp90, was even shown to play an active role in promoting the release of exosomes in a *Drosophila* model system [44]. The secreted Hop and its cytokine-like functions in CNS development and cancer have recently been reviewed [45]. Here, we will focus on the intracellular and physiological functions of Hop.

## Structure of Hop

**Fig. 1:**
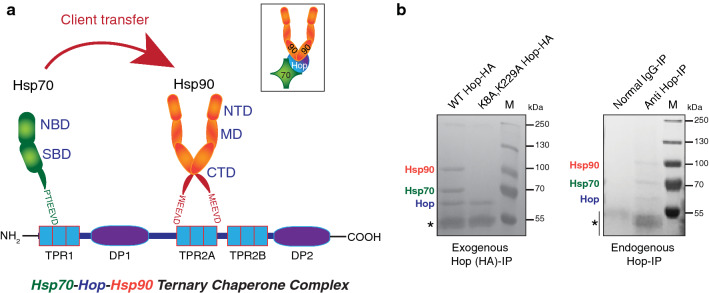
The Hsp70-Hop-Hsp90 ternary molecular chaperone complex. **a** A schematic representation of the domain structure of Hop and of the mode of interaction of Hop with Hsp70 and Hsp90 in forming the Hsp70-Hop-Hsp90 ternary chaperone complex. Inset: a simplified model of the ternary chaperone complex used in subsequent figures. **b** Hsp70 and Hsp90 are the major interactors of both exogenously expressed and endogenous Hop. The images are of Ponceau S-stained membranes of immunoprecipitation experiments (IP) as indicated. TPR domain double mutant (K8A (TPR1), K229A (TPR2A)) Hop serves as a negative control (left panel) [28]. Normal mouse IgG serves as a control for the endogenous Hop immunoprecipitation (right panel). The position of immunoglobulin heavy chains is marked with an asterisk

A prediction of the Sti1 protein structure was first proposed back in 1989 along with its discovery [17]. Its predicted amino acid sequence showed it to be a rather hydrophilic protein [17]. Secondary protein structure prediction indicated that most of the regions of Sti1 exist in α-helical domains [17]. Sti1/Hop contains three tetratricopeptide repeat (TPR) domains and two aspartate-proline-rich polypeptide (DP) segments [16, 19, 46] (Fig. 1a). TPR domains comprise three or more TPR motifs (tandem array of two antiparallel α‐helices), and each motif consists of 34 amino acid repeats [47]. The TPR domains of Sti1/Hop are called TPR1, TPR2A, and TPR2B, while the two DP domains are named DP1 and DP2 [48]. Overall, structurally, the TPR domains are highly organized α-helices [47]. The TPR1, TPR2A, and TPR2B domain structures of yeast and human Sti1/Hop were evaluated either by X-ray diffraction or solution NMR [16, 49–51]. These studies experimentally confirmed the predicted α-helical structures of the TPR domains of Hop. The DP domains were considered to be unstructured until solution NMR spectroscopy revealed highly homologous α‐helical folds with six helices in DP1 and five helices in DP2 of Sti1 [49]. While the individual domain structures of yeast and human Sti1/Hop are known, the structure of full-length Sti1/Hop has yet to be solved. Several studies reported Hop to be a dimeric protein [52–55]; however, this was contradicted by others claiming that Hop is a monomeric protein [56, 57]. A cryo-EM analysis of a Hop-Hsp90 complex suggested that a Hsp90 dimer binds only one Hop molecule [58].

## Hsp70 and Hsp90: the major interactors of Hop

Hop was first discovered as one of the major interactors of Hsp90 by immunoaffinity purification of Hsp90 from chicken oviduct cytosol [46]. This Hsp90-Hop complex was found to be simultaneously associated with Hsp70. This finding strongly suggested the existence of a Hsp70-Hop-Hsp90 ternary complex in the eukaryotic cytosol (Fig. 1a). Although immunoaffinity purification of Hsp90 co-precipitates substoichiometric amounts of Hsp70 and Hop [46], we recently found that the use of an antibody to (HA-tagged) Hop co-immunoprecipitates almost equimolar amounts of Hsp70 and Hsp90 from HEK293T cells [28] (Fig. 1b). This indicated that most of the intracellular Hop molecules are complexed with Hsp70 and Hsp90 [28]. However, evidence has been emerging that intracellular Hop can interact with other proteins independently of its binding to either Hsp70 or Hsp90 or both [59–64].

What is the molecular basis for the simultaneous interaction of Hop with Hsp70 and Hsp90? The TPR1 domain of Hop is responsible for the binding to the extreme C-terminal heptapeptide PTIEEVD of Hsp70 (Fig. 1a). TPR2A serves as a high-affinity binding site for the extreme C-terminal peptide MEEVD of Hsp90 [16] (Fig. 1a). The conserved EEVD motifs at the C-termini of Hsp70 and Hsp90 behave like a dicarboxylic acid clamp and are responsible for the electrostatic interactions with the respective TPR domains of Hop [16, 49, 65–67]. The selectivity and specificity of binding of the respective TPR domains to the C-termini of Hsp70 and Hsp90 are conserved between humans and yeast [16, 49].

Unlike TPR1 and TPR2A, the TPR2B domain of Sti1/Hop can bind both Hsp70 and Hsp90 without a marked preference for either one of the C-termini, but its binding is of relatively low affinity [49]. Thus, Sti1/Hop has one high-affinity binding site each for Hsp90 (TPR2A) and Hsp70 (TPR1), and an additional, less selective Hsp70/Hsp90 binding site (TPR2B). Hsp70 may initially bind to the TPR1 domain of Sti1/Hop in an open conformation, and then transfer to TPR2B once Hop binds Hsp90 via TPR2A [68]. Moreover, additional contact sites with the CTD and MD of Hsp90 are also evident [49, 55, 69–71].

Different biophysical techniques, including surface plasmon resonance, isothermal calorimetry, analytical ultra-centrifugation, hydrogen exchange mass spectrometry, and luminescence resonance energy transfer, have been used to determine and characterize the affinities [dissociation constants (*K*_D_)] of the respective TPRs of Hop for the C-terminal sequences of Hsp70 and Hsp90 [16, 48, 49, 72, 73]. Tomm34, another multiple TPR domain-containing protein and Hsp90 co-chaperone of the outer mitochondrial membrane, has also been shown to bind simultaneously to Hsp70 and Hsp90 [74]. However, Hop cannot be functionally substituted by Tomm34, most notably in Hop knockout cells because the abundance of Tomm34 is too low [28]. Although it appears that Hop cannot be functionally replaced in the Hsp70–Hsp90 chaperone complex by another TPR-containing protein, Hop competes for Hsp90 binding with several other TPR domain-containing proteins, such as cyclophilins, including FK506 binding proteins (FKBPs) or peptidyl-prolyl isomerase (PPIase), and Stub1/Chip [75–77]. In fact, there is evidence that mixed complexes such as Hsp90-FKBP52-Hop-Hsp70, Hsp90-FKBP52-p23-Hop, and Hop-PPIase-Hsp90 also exist in cells, albeit in reduced proportions [54, 57, 77, 78]. It was proposed that serine/threonine phosphorylation just N-terminal to the EEVD motifs of the C-termini of Hsp70 and Hsp90 enhances the binding to Hop and prevents the binding to Stub1/Chip [79]. Posttranslational modifications on either Hsp70 or Hsp90 or the co-chaperones influence interaction preferences and need to be studied more thoroughly in the future.

## Impact of Hop on the Hsp70–Hsp90 chaperone cycle and client maturation

**Fig. 2:**
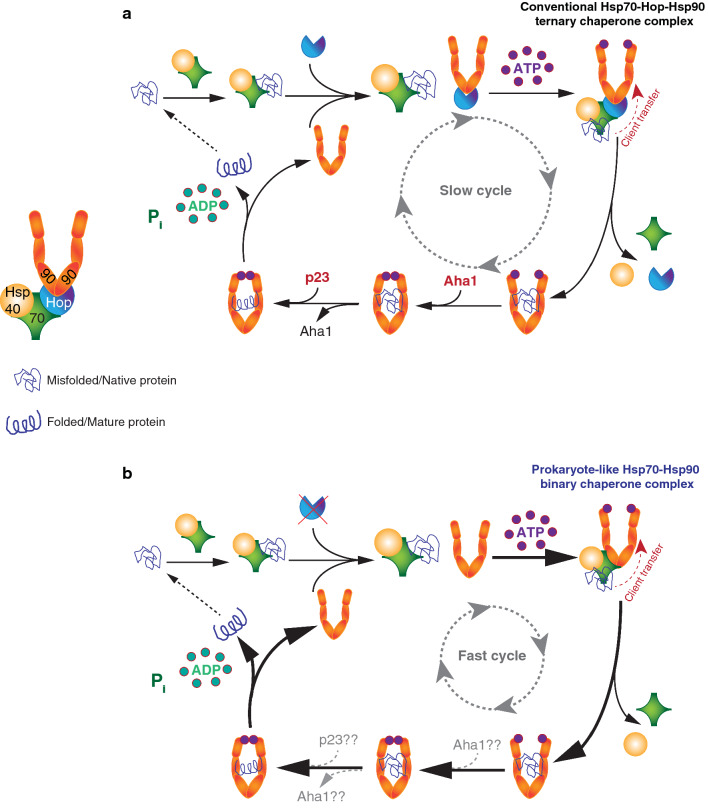
Schematic representation of the two alternate Hsp90 chaperone cycles. **a** Canonical Hsp90 chaperone cycle driven by the Hsp70-Hop-Hsp90 ternary chaperone complex. **b** Proposed model for the alternate Hsp90 chaperone cycle in the absence of Hop. The prokaryote-like Hsp70–Hsp90 binary chaperone complex drives the alternate Hsp90-mediated protein folding activities

Hsp70 and Hsp90 are the evolutionarily conserved molecular chaperones that assist in the folding of nascent polypeptides and stress-induced misfolded proteins in the cytosol [4, 10]. Substrates are recognized by Hsp70 and a J-domain containing Hsp70 co-chaperone, typically Hsp40 in eukaryotes [5, 80, 81] (Fig. 2a, b). J-domain protein (JDPs) such as Hsp40 stimulate the Hsp70 ATPase activity and enhance the substrate binding affinity of Hsp70 by promoting its conversion to the ADP-bound state [82]. The NEF-dependent ADP release from Hsp70 allows the binding of ATP, which triggers its substrate release [1]. Although Hsp70 was thought to be a solitary molecular chaperone for protein folding and refolding, the hydrophobic properties of its substrate-binding cavity could even arrest folding for some substrates [4]. A recent finding experimentally supports the idea of Hsp70 acting as a preventer of protein folding. It was discovered that Hsp70, when tested at physiological concentrations in vitro, inhibits the folding of a model substrate [83]. Because of its hydrophilic nature, Hsp90 does not block protein folding as Hsp70 does [4, 83]. Thus, Hsp90 becomes essential to break this “deadlock” of the Hsp70 chaperone system by allowing the completion of the protein folding process and the subsequent release of active substrates [83]. As a result, Hsp70 and Hsp90 functionally collaborate to enhance the yield of folded proteins [83] (Fig. 2a, b).

In eukaryotic cells, the cooperation between Hsp70 and Hsp90 is mediated by Hop, as discussed above. Formation of the Hsp70-Hop-Hsp90 ternary chaperone complex may be the rate-limiting step for the substrate transfer from the Hsp70 system to the Hsp90 system [68] (Fig. 2a). Upon substrate binding, Hsp90 undergoes large conformational changes concerted with the sequential and highly regulated binding of other co-chaperones, including Aha1, PPlase, and p23 [10, 12, 84, 85] (Fig. 2a). Aha1 binds to the MD of Hsp90 and stimulates its ATPase activity, which is a prerequisite for substrate protein folding and subsequent release [86]. Two molecules of p23 bind to the NTDs of the Hsp90 dimer in its closed state and facilitate the final steps of substrate-protein folding, maturation, and release in a concerted manner with Aha1 [87–90] (Fig. 2a). After completion of a successful ATPase cycle, the Hsp90 dimer transforms into an open conformation and is restored for the next chaperone cycle (Fig. 2a). Each co-chaperone acts uniquely on the ATPase activity of Hsp90 and the conformational dynamics of the entire Hsp90 dimer [10, 12, 84, 85, 91].

Hop is the physiological allosteric inhibitor of the ATPase activity of Hsp90 [53, 57, 65]. Hop does so probably by stabilizing the open conformation of the Hsp90 dimer and blocking the N-terminal closure of Hsp90, which is required for its ATPase activity [65]. Although Hop blocks the ATPase activity of Hsp90, it does not inhibit ATP binding to the NBD of Hsp90 [65]. TPR2A-TPR2B is the minimally required segment of Hop for maximum inhibition of the ATPase activity of Hsp90 [48, 49]. TPR2B of Hop/Sti1 occupies a position at the MD of Hsp90, between the two protomers of the Hsp90 dimer, thereby sterically restricting the conformational rearrangement of the MD and NTD closure during the ATPase cycle [49]. A cryo-EM analysis showed that the TPR1 domain of Hop could also sterically block the Hsp90 N-terminal dimerization by being situated between the Hsp90 protomers and interacting with the adjacent NTD and MD [58].

By itself, the TPR2A–TPR2B domains of Hop are unable to facilitate the activation of Hsp90 clients in vivo despite the fact that this portion of Hop retains the full inhibitory capacity for the ATPase activity of Hsp90 [49]. For functionality it needs DP2. The TPR2A–TPR2B–DP2 portion of Hop is the minimally required functional module to recapitulate most of the in vivo activities of full-length Hop for Hsp90 client protein folding and maturation [49]. Remarkably, the TPR1 domain of Hop was proposed to be dispensable for Hsp90-client protein folding [49]. This is further supported by the fact that the *Caenorhabditis elegans* Hop ortholog lacks a TPR1 domain and yet is fully functional [92–94]. In contrast to the inhibitory effect of Hop on the ATPase activity of Hsp90, Sti1 binding enhances the ATPase activity of Hsp70, thereby promoting the activity of Hsp70 in yeast [95]. Human Hop is unable to activate the ATPase of human Hsp70 since its basal ATPase activity is higher to begin with [95]. A genetic analysis of the determinants of Sti1-dependence of Hsp90 in budding yeast yielded a wealth of Hsp90 point mutants [96]. Overall, the results are also consistent with Sti1 promoting the recruitment of client-loaded Hsp70 and client transfer through a conformational change of Hsp90 favoring its N-terminal closure [96].

Little is known about the relevance of posttranslational modifications of Hop for Hsp70–Hsp90 functions. A study regarding a few phosphorylation sites of human and yeast Hop revealed that, in general, phosphorylation of Hop is counterproductive for the Hsp70–Hsp90-driven client protein folding and maturation processes [97, 98] (https://www.phosphosite.org/proteinAction.action?id=2638&showAllSites=true). Further studies are warranted to clarify additional functional modifications of Hop, the upstream regulators, and their relevance for cellular physiology and disease pathology.

One interesting question is whether Hop by itself can act as a molecular chaperone. Efficient conversion of chemical energy into mechanical energy was thought to be the prerequisite for a protein to be a molecular chaperone. Recently, this idea has been experimentally proven for the Hsp70 chaperone system [99]. Although Hop was reported to bind ATP and to have a slow ATPase activity [100], Hop is unable to fold substrates by itself [52, 101]. Therefore, since Hop fails to fulfill the criteria for being a molecular chaperone, it must be considered a co-chaperone of the Hsp70–Hsp90 chaperone system.

## Hsp70-Hop-Hsp90 ternary complex versus Hsp70–Hsp90 binary complex

Hop appeared during the evolution of eukaryotic organisms [17, 102] whereas no Hop-like protein has been discovered in bacteria. Bacterial Hsp70 (DnaK) and Hsp90 (HtpG) must, therefore, be able to interact directly during substrate transfer [103–107]. The *Escherichia coli* (*E. coli)* Hsp70–Hsp90 binary complex is further stabilized by the interaction with both clients and a DnaJ protein, the bacterial JDP [104]. Mutating surface residues that are essential for the direct interaction between *E. coli* Hsp70 and Hsp90 impair binary complex formation and prevent substrate protein folding [104]. A recent computational model places the client proteins bound to the SBD of bacterial Hsp70 in the proximity of the client-binding site of bacterial Hsp90, which may facilitate substrate transfer from Hsp70 to Hsp90 [105]. The direct interaction of these bacterial molecular chaperones mutually and synergistically enhances their ATPase activity in the presence of substrate proteins [104–106].

The appearance of Hop in eukaryotes correlates with the modification of the C-termini of eukaryotic cytosolic Hsp70 and Hsp90, such that they contain the extreme C-terminal EEVD motif allowing Hop binding (Fig. 1a). And yet, recent discoveries show that eukaryotic Hsp70 and Hsp90 can interact directly in the absence of Hop both in vitro and in vivo [28, 108] (Fig. 2b). This Hop-independent Hsp70–Hsp90 binary complex is functionally active for substrate folding/refolding and even with a higher rate and yield [28] (Fig. 2b). Since Hop is a physiological inhibitor of the ATPase activity of Hsp90, we proposed that the Hsp70–Hsp90 binary complex has a higher folding capacity than the Hsp70-Hop-Hsp90 ternary complex. This may be the case, at least for some substrates, because the binary complex is not restrained anymore by Hop (Fig. 2b) [28].

Conserved surface residues that are essential for the direct interaction of bacterial Hsp70 and Hsp90 are also essential for the eukaryotic Hsp70–Hsp90 binary complex formation and for substrate refolding [28]. Therefore, Hsp70 and Hsp90 retain their ability to form a more ancient prokaryote-like functional chaperone complex even in eukaryotes (Fig. 2b). Further details of the functional aspects of these binary and ternary molecular chaperone complexes are discussed in the following sections.

The endoplasmic reticulum and mitochondria, which have paralogs of Hsp70 and Hsp90, lack Hop or Hop-like proteins. Their Hsp70 and Hsp90 physically and functionally interact directly [109, 110, see also ref. 111] and also lack the extreme C-terminal EEVD motif. Whether Hsp70 and Hsp90 can interact directly in the eukaryotic cytosol in the presence of Hop and whether this alternative binary chaperone complex has any specialized functions are still open questions. *Caenorhabditis elegans* could be an interesting model organism to test this notion since its Hop lacks the TPR1 domain and competitively interacts with Hsp70 and Hsp90 [92, 93]. For *Caenorhabditis elegans*, it remains to be determined whether Hsp70 and Hsp90 interact directly or whether the TPR2A-TPR2B domains of Hop are sufficient to form a functional Hsp70-Hop-Hsp90 ternary complex.

## Phenotypes of Hop mutants in eukaryotic organisms

Since Hop is specifically present only in eukaryotic organisms, and strictly required for the client transfer to Hsp90 and folding in some biochemical assays, Hop was thought to be indispensable for eukaryotic life. Therefore, deletion or dominant-negative mutations of the Hop encoding genes could be expected to be lethal. Contradicting this speculation, we now know that budding yeast, worms (*Caenorhabditis elegans*), flies (*Drosophila melanogaster*), and human cancer cell lines are viable without Hop [17, 28, 93, 112]. Whereas *∆sti1* yeast strains are viable, but heat- and cold-shock sensitive [17]. These mutant yeast strains as well as human cancer cells lacking Hop are somewhat more dependent on Hsp90 functions since they are hypersensitive to Hsp90 inhibitors [28, 113–116]. *∆sti1* strains are also hypersensitive to molybdate, which stabilizes Hsp90 in the closed conformation even after ATP hydrolysis and is thought to constrain the dynamic rearrangements of Hsp90 [117]. This agrees with the fact that deletion of either *hsc82* or *hsp82* is synthetically lethal with *∆sti1* in yeast under normal growth conditions or at elevated temperature, respectively [76]. In line with these notions, a series of temperature-sensitive mutations in the MD of yeast Hsp90, which are defective for the interaction with yeast Hsp70, have been shown to be synthetically lethal with *∆sti1*[108]. Several other mutations in yeast have been discovered to be synthetically lethal with *∆sti1* including *cpr7, cdc37, ydj1, mps1, sse1, sba1, ssl2, emc2, and utp21* [63, 116, 118–123].

*Caenorhabditis elegans* is viable in the absence of Hop, but the null mutant has decreased fertility, a shortened lifespan, and an impaired heat-shock response [93]. Unlike yeast and worm models, several Hop knockout human cancer cell lines are either equally or rather less sensitive to acute heat shock compared to their wild-type counterparts [28]. One can imagine that the relatively hyperactive Hsp70–Hsp90 binary complex in the absence of Hop might be protective upon heat shock. In contrast, the knockdown of Hop in human HEK293T cells with a doxycycline-inducible system was reported to reduce viability under basal and heat-shock conditions [124]. These differences between the knockout and knockdown models are striking and may be explained by the long-term adaptation to the Hop depletion during the clonal establishment of Hop knockout cells. This apparent discrepancy will have to be resolved methodically in the future. The sensitivity to other types of stresses [125–127] may also depend on the exact experimental conditions and on cell type, and will require further investigations.

Unlike the above-mentioned models of Hop mutants, Hop appears to be essential for mouse embryonic development [125] and for the viability of *Leishmania donovani* promastigotes [128]. Hop knockout mouse embryos die at the stage of E10.5; the knockout causes improper neural tube formation, increased DNA damage and stress susceptibility, and apoptosis, which may all contribute to embryonic lethality [125]. Even heterozygotes display increased cellular stress and are more sensitive to cerebral ischemia [125]. Mice with a Hop TPR1 truncation can be born, but they have a high mortality rate, possibly because the truncation mutant accumulates to considerably lower levels [126]. These mouse models clearly demonstrate that not every eukaryotic cell may be able to shift from the Hsp70-Hop-Hsp90 ternary complex to the Hsp70–Hsp90 binary complex functionally, and that even a reduced level of functional Hop is detrimental to the adult life in mouse. It has been proposed that the absence of extracellular and not just intracellular Hop in these mouse models might contribute to the lethality of neuronal cells [125]. However, the details of the molecular targets and mechanisms behind the death of the mouse embryos lacking Hop warrant further investigations.

## Hop as a regulator of proteostasis

Proteostasis is the overall process by which the cellular proteome is maintained in a functional state. All axes of proteostasis, including protein translation, folding/refolding, and degradation, are extensively regulated by molecular chaperones [129–132]. Both Hsp70 and Hsp90 are collectively involved in ribosomal biogenesis, translation, and co-translational protein folding [133–138]. Multidomain cytosolic proteins typically undergo chaperone-assisted protein folding by Hsp70 and Hsp90. Hsp90 can also transiently hold structurally labile or misfolded proteins to prevent their aggregation [139], similarly to how small Hsps use their “holdase” function [140]. Intriguingly, Hsp70 and Hsp90 are not only responsible for protein synthesis and stability, but they also regulate protein degradation. Hsp70 is the master regulator of chaperone-mediated autophagy (CMA) of degradation-prone substrate proteins carrying a KFERQ motif as a recognition peptide signal sequence [130]. With the assistance of its co-chaperone proteins, Hsp70 delivers the substrate proteins to lysosomes via an interaction with a lysosomal receptor LAMP2A [6, 7]. Although Hsp90 and its co-chaperones were also proposed to be involved in the CMA process, the molecular basis has yet to be revealed [7, 141].

Hsp90 is involved in protein degradation by the proteasome [28, 142]. Hsp90 was proposed to be a regulator of proteasome assembly and stability, and to facilitate proteasome reassembly after a heat shock-induced disassembly in yeast [142]. In yeast, mutations in several proteasomal genes were found to be synthetically lethal with *hsp82* and *hsc82* mutations [142]. Intriguingly, co-expression of Hsp90 is essential for the efficient formation of the recombinant proteasomal 19S lid complex in bacteria [143]. Several proteasome-dedicated chaperones are known for the 20S catalytic core and 19S base assembly, only Hsp90 has been proposed as a chaperone for 19S lid assembly to date [143]. Beyond the putative function of Hsp90 in lid assembly, for specific substrate proteins, Hsp90 serves as a chaperone for direct loading into the free 20S proteasomal core complex for degradation [144]. Thus, the Hsp70 and Hsp90 molecular chaperone systems are not only protein folding hubs, but they are also involved in many other aspects of proteostasis.

**Fig. 3:**
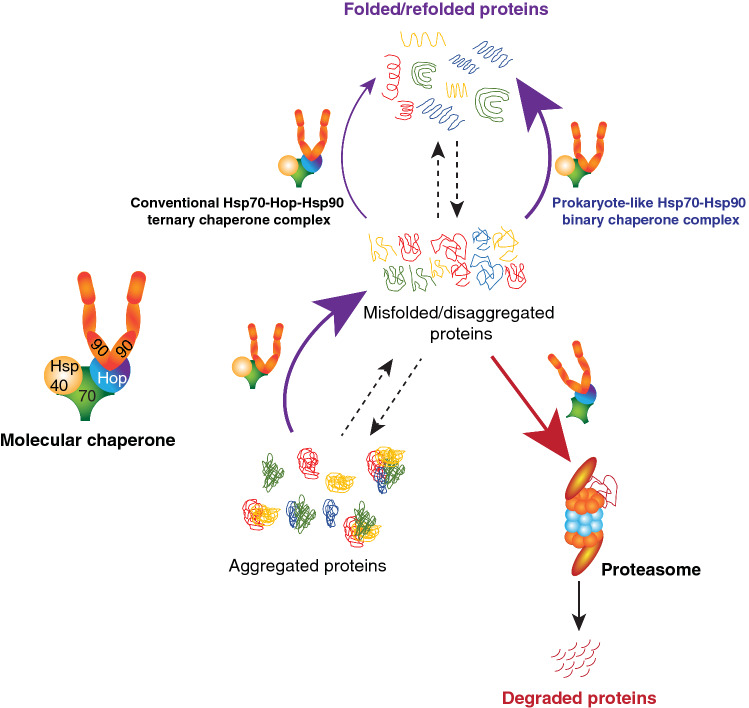
Schematic representation of the impact of Hop, together with Hsp70 and Hsp90, on both arms of proteostasis (protein folding and degradation)

*Hop in protein degradation* We recently found that Hop promotes proteasome assembly and protein degradation [28] (Fig. 3), and that this novel function of Hop is evolutionarily conserved between humans and yeast [28]. The ability of Hop to form the Hsp70-Hop-Hsp90 ternary chaperone complex is essential for this function [28] (Fig. 3). Hop TPR domain mutants, that can neither bind to Hsp70 nor Hsp90 nor both are unable to rescue the defects of proteasomal assembly and protein degradation in Hop knockout cells [28]. We proposed that the Hsp70-Hop-Hsp90 ternary chaperone complex chaperones the docking of the 19S regulatory complex of the proteasome to the 20S core [28]. We further suggested that the Hsp70-Hop-Hsp90 ternary complex may contribute to stabilizing and maintaining the 26S/30S proteasome through transient regulatory interactions with some proteasomal subunits [28]. The molecular basis for the functions of the Hsp70-Hop-Hsp90 ternary complex in proteasome assembly and stability are not completely understood yet. Since the full atomic structure of the eukaryotic proteasome is available [145–147], experimental mapping of the Hsp70-Hop-Hsp90 ternary complex on the structures of proteasome subcomplexes or of the fully assembled mature proteasome by cryo-EM could be highly instructive.

In the context of the functions of the Hsp70-Hop-Hsp90 ternary chaperone complex in proteasomal assembly and function, there are several other open questions: (1) whether and how the ATP binding ability and ATPase activities of Hsp70 and Hsp90 are required; (2) whether other co-chaperones of the Hsp70 and/or Hsp90 chaperone systems are involved; (3) whether there are posttranslational modifications in any of the proteins of the ternary chaperone complex that may influence this process; (4) whether the Hsp70-Hop-Hsp90 complex collaborates with the canonical proteasome assembly chaperones to achieve the highest yield in the formation of the 26S/30S proteasome.

*Hop in protein folding* Surprisingly, protein folding appears to be even more efficient in the absence of Sti1/Hop in both yeast and human cancer cell lines [28] (Fig. 2b). The prokaryote-like binary complex consisting of Hsp70 and Hsp90 cannot only form in eukaryotic organisms in the absence of Hop, but this alternative chaperone complex is fully functional and ensures proteostasis [28]. As with the *E. coli* proteins, Hsp70, Hsp90, a JDP, a NEF, and ATP are the minimally required components for the in vitro refolding of the model substrate luciferase by the human Hop-independent alternative Hsp70–Hsp90 chaperone complex [28] (Fig. 2b). Increasing concentrations of Hop gradually decrease refolding of luciferase in vitro, and the direct interaction between Hsp70 and Hsp90 is essential for the refolding activity since mutating the interaction surface of Hsp90 reduces it [28]. The situation seems to be somewhat different with the components from budding yeast since the protein folding yield is positively influenced by Sti1 even though the Hsp70 and Hsp90 orthologs can also interact directly [108]. Several experimental differences may explain this apparent discrepancy: (1) As mentioned above, Sti1 and Hop affect the ATPase activity of their respective Hsp70 differently [95]; (2) different absolute or relative protein concentrations may have been used in these studies, which may be critical considering that different concentrations of Hsp70 can differentially impact the final yield of folded protein in these in vitro assays [4, 83].

While the Hsp70–Hsp90 binary complex, in the absence of Hop, is sufficient for folding and maturation of most Hsp90 clients in vivo, there are a few notable exceptions, including the glucocorticoid receptor (GR), v-Src, and, of course, the proteasome [28, 148]. Reduced levels of Hop have also been reported to affect the accumulation or stability of a few other Hsp90 clients, including LSD1 [149], GRK2 and Tau [126], TDP-43 [127], and MMP2 [150] in various cellular and mouse models. It could be hypothesized that Hop may influence client selectivity of Hsp90 in eukaryotic organisms [28, 148]. Intriguingly, for some Hsp90 clients, in vivo and in vitro systems yield differential protein folding and functional activities. For instance, the transfer of p53 from Hsp70 to Hsp90 and the folding to its native state require Hop in vitro [151, 152]. In contrast, in human cells, p53 protein levels are unaffected and its transcriptional activity is either the same or enhanced in the absence of Hop [28]. These discrepancies suggest that in the more complex intracellular milieu, the Hsp70–Hsp90 binary complex might be supported by other co-chaperones of the Hsp70 and Hsp90 systems, and/or yet other proteins (Fig. 2b). A genome-wide screen with the CRISPR-Cas9 system might reveal additional essential players responsible for the maximum chaperoning capacity of the Hsp70–Hsp90 binary complex. All of this evidence supports the conclusion that Hop is a novel regulator of proteostasis, whose levels and activity determine the balance between protein folding and degradation (Fig. 3).

## Hop in cancer and neurodegenerative disorders

In cancer cells, Hsp90 is upregulated, and many oncogenic proteins (for example c-Abl, c-Raf, b-Raf, c-Src, Cdks, cyclins, mutant p53, Stat3, steroid receptors) rely on the chaperoning functions of Hsp90 for their folding, maturation, and activation [153–156]. This has led to the conclusion that Hsp90 is an oncogenic molecular chaperone [154, 155]. Similarly, Hsp70 is upregulated in cancer and favors oncogenesis [157, 158]. It has been reported that cancer types with more integrated and connected Hsp90-centered proteome hubs ("epichaperome"), of which Hop is an integral part, are more sensitive to Hsp90 inhibition than those with insular Hsp90 complexes [159, 160]. Consistent with the upregulation of both Hsp70 and Hsp90, Hop is also found to be overexpressed in several cancers at both the mRNA and protein levels [36–39, 161, 162]. These findings are reminiscent of how Hop was first discovered in human cells (see above) [19]. It was initially designated "transformation-sensitive human protein" IEF SSP 3621, before it was recognized that it is the human ortholog of the yeast protein Sti1 [19]. Although Hop is upregulated during oncogenesis, whether its overexpression is the consequence of cancer or whether it can initiate cancer, or, in other words, whether the Hop gene *STIP1* is an oncogene still needs to be clarified.

Reducing Hop levels by RNA interference can revert cancer-specific phenotypes, including proliferation, invasion, migration, and metastasis [61, 150, 162, 163]; it reduces the levels of several Hsp90 clients, which correlates with the anticancer effects [162, 163]. While the outright knockout of Hop in human cancer cell lines, as mentioned above, minimally affects Hsp90 clients, it does further sensitize cancer cells to Hsp90 inhibitors [28]. Further studies are necessary to resolve these apparent differences between Hop knockout and knockdown models.

Unlike in cancer, all axes of the proteostatic network are reduced in some neurodegenerative disorders, including the levels of Hsp70 and Hsp90, as judged by their mRNA levels [164]. On the contrary, for Alzheimer's disease, the evidence suggests that the proteins Hsp70, Hsp90, and Hop are all upregulated [165–167]. Aggregation of certain pathological proteins correlates with the pathogenesis and prognosis in neurodegeneration. Many pathogenic proteins, which are thought to be responsible for neurodegenerative diseases, are Hsp90 clients or interactors, including tau (Alzheimer’s disease), α-synuclein (αSyn, Parkinson’s disease), and huntingtin (Htt, Huntington’s disease). A key question is whether it is the large insoluble aggregates or the soluble oligomers that are the most pathogenic. Although there is evidence in support of both hypotheses, most would agree today that it is the soluble oligomers, which are toxic to neurons [168, 169], and that therapeutic strategies should focus on them.

The chaperoning functions of Hsp70 are considered to be neuroprotective [164, 170], whereas the situation is confusing for Hsp90 and other components of the Hsp90 complex. Hsp90 has been proposed to inhibit the formation of oligomeric αSyn, further aggregation of which disrupts the mitochondrial membrane resulting in impaired mitochondrial function and the subsequent neuronal death in Parkinson’s disease [171]. For tau-toxicity in Alzheimer’s disease, Hsp90 has the opposite effect as it stabilizes the toxic soluble oligomers of tau in collaboration with its co-chaperones FKBP51/52 and Aha1; in this case, it prevents the formation of the cytoprotective larger aggregates [172–174]. The Hsp90 epichaperome was also proposed to be involved in the loss of dopaminergic neurons in the midbrain in Parkinson’s disease [175]. Therefore, directly manipulating the Hsp90 levels or activity might produce an opposite outcome depending on the underlying molecular mechanisms of a given neurodegenerative disease.

Relatively little is known about the functions of intracellular Hop in neurodegenerative diseases. Intracellular Hop in yeast protects cells from the toxicity of a mutant huntingtin with an aggregation-prone stretch of 103 glutamine residues (Htt103Q), and of the (PSI^+^) prion [176, 177]. In *Caenorhabditis elegans*, the knockdown of Hop aggravates the protein aggregation toxicity of amyloid β42 (Aβ42) without affecting lifespan [164]. In *Drosophila melanogaster,* loss of function of Hop enhances the toxicity of exogenous human tau [112]. Murine Hop, together with Hsp90, significantly reduces the aggregation of TDP-43, whose misfolding and formation of inclusions are hallmarks of amyotrophic lateral sclerosis [127]. Interestingly, as alluded to above, an age-dependent upregulation of Hop is evident in a mouse model of Alzheimer’s disease and in Alzheimer’s disease-affected human patients [167]. In all of these neurodegenerative disease models Hop seems to be protective, but several examples with opposing effects have emerged, too. A recent genetic screen in *Drosophila melanogaster* discovered beneficial effects of reduced levels of Hop*.* A Hop knockdown reduces the aggregation of Htt mutants with polyglutamine expansions and prevents their toxic phenotype [178]. Reminiscent of this finding, we recently demonstrated that the knockout of Hop in human cells reduces the aggregation of Htt72Q [28] (Fig. 3). The increased anti-aggregation activity of these cells requires Hsp90, since the chemical inhibition of Hsp90 reversed the phenomenon [28]. Intriguingly, the overexpression of Hop in a particular mouse model of Alzheimer’s disease has been reported to increase the accumulation of Aβ [179]. This evidence raises the possibility that molecular chaperones and their co-chaperones might only be neuroprotective within a narrow concentration range. Overall, the molecular and pathological functions of Hop in neurodegenerative disorders remain confusing and need to be explored more thoroughly using several more comparable disease-specific models. Manipulating the levels or activities of Hop might eventually be a promising avenue to treat both cancer and neurodegeneration.

## Conclusions and perspectives

Studies on Hop have revealed its importance in fundamental cellular processes in both normal and pathophysiological conditions. Hop is differentially expressed in diseases like cancer and in aging, and it critically regulates activities of the Hsp70–Hsp90 chaperone system. Hop might, therefore, be an attractive target for future personalized medicine approaches.

Hsp90 inhibitors have been the subject of many clinical trials [154, 180] as anticancer therapies because cancer cells are more sensitive to such inhibitors than normal cells (discussed in [181]). The current knowledge, translational difficulties, and future perspectives of targeting Hsp90 in cancer have recently been reviewed [182–184]. Despite the initial setbacks of targeting Hsp90 in cancer, their addiction to Hsp90 may justify renewed efforts. These will include the more specific targeting of cancer-relevant isoforms of the Hsp90 family. Inhibiting Hsp90 co-chaperones rather than the whole Hsp90 chaperone machinery might also be worth exploring [28, 116, 185, 186]. Since *∆sti1* yeast mutants [113–116] and human Hop knockout cancer cells [28] are hypersensitive to Hsp90 inhibitors, combining Hop and Hsp90 inhibitors may be promising as cancer therapy. The inhibition of Hop would be expected to potentiate the effects of Hsp90 inhibitors, which could then be used at lower and even more cancer-specific doses. Those types of cancer that rely both on Hsp70–Hsp90-mediated protein folding and proteasome-mediated protein degradation may be particularly vulnerable since the inhibition of Hop reduces the proteasomal activity, which itself is an already established anti-cancer target. Some compounds have been demonstrated to inhibit the interaction between Hsp90 and the TPR2A domain of Hop [51, 187, 188]. The challenge will be to develop inhibitors that are as specific as possible for the interaction of Hop with either Hsp70 or Hsp90, without affecting the interactions of the latter with other TPR-containing co-chaperones.

Although Hsp90 function in neurodegenerative disorders remains paradoxical, as described above, activation of the chaperoning functions of the Hsp70–Hsp90 chaperone system could be therapeutically beneficial at least in some cases [164, 189]. Augmenting the activity of a protein or an enzyme is always challenging, but our recent findings revealed that the genetic removal of Hop strikingly enhances protein folding by the Hsp70–Hsp90 binary complex (Figs. 2b, 3) and reduces the aggregation of the pathological huntingtin protein [28] (Fig. 3). It remains to be seen whether non-toxic Hop inhibitors can be developed to boost Hsp70–Hsp90 chaperoning, notably in neuronal cells, and whether these inhibitors could be applied to certain neurodegenerative disorders.

## Data Availability

Not applicable.
